# Genome wide identification of GDSL gene family explores a novel *GhirGDSL*26 gene enhancing drought stress tolerance in cotton

**DOI:** 10.1186/s12870-022-04001-0

**Published:** 2023-01-07

**Authors:** Jiajun Liu, Jiangna Liu, Heng Wang, Aziz Khan, Yanchao Xu, Yuqing Hou, Yuhong Wang, Zhongli Zhou, Jie Zheng, Fang Liu, Xiaoyan Cai

**Affiliations:** 1 State Key Laboratory of Cotton Biology, Institute of Cotton Research, Chinese Academy of Agricultural Sciences, Anyang, 455000 China; 2 Key Laboratory of Plant Genetics and Breeding, College of Agriculture, Guangxi University, 530005 Nanning, China; 3 Hainan Yazhou Bay Seed Laboratory, Sanya, 572024 China; 4 National Nanfan Research Institute (Sanya), Chinese Academy of Agriculture Sciences, Sanya, 572025 China; 5 School of Agricultural Sciences, Zhengzhou University, Zhengzhou, China

**Keywords:** Cotton, *GDSL* gene, Drought resistance, Functional identification

## Abstract

**Background:**

Current climate change scenarios are posing greater threats to the growth and development of plants. Thus, significant efforts are required that can mitigate the negative effects of drought on the cotton plant. GDSL esterase/lipases can offer an imperative role in plant development and stress tolerance. However, thesystematic and functional roles of the GDSL gene family, particularly in cotton under water deficit conditions have not yet been explored.

**Results:**

In this study, 103, 103, 99, 198, 203, 239, 249, and 215 GDSL proteins were identified in eight cotton genomes i.e., *Gossypium herbaceum* (A1), *Gossypium arboretum* (A2), *Gossypium raimondii* (D5), *Gossypium hirsutum* (AD1), *Gossypium barbadense* (AD2), *Gossypium tomentosum* (AD3), *Gossypium mustelinum* (AD4), *Gossypium darwinii* (AD5), respectively. A total of 198 *GDSL* genes of *Gossypium hirsutum* were divided into eleven clades using phylogenetic analysis, and the number of *GhirGDSL* varied among different clades. The *cis*-elements analysis showed that *GhirGDSL* gene expression was mainly related to light, plant hormones, and variable tense environments. Combining the results of transcriptome and RT-qPCR, *GhirGDSL26* (*Gh_A01G1774*), a highly up-regulated gene, was selected for further elucidating its tole in drought stress tolerance via estimating physiological and biochemical parameters. Heterologous expression of the *GhirGDSL26* gene in *Arabidopsis thaliana* resulted in a higher germination and survival rates, longer root lengths, lower ion leakage and induced stress-responsive genes expression under drought stress. This further highlighted that overexpressed plants had a better drought tolerance as compared to the wildtype plants. Moreover, 3, 3’-diaminobenzidine (DAB) and Trypan staining results indicated reduced oxidative damage, less cell membrane damage, and lower ion leakage in overexpressed plants as compared to wild type. Silencing of *GhirGDSL26* in cotton via VIGS resulting in a susceptible phenotype, higher MDA and H_2_O_2_ contents, lower SOD activity, and proline content.

**Conclusion:**

Our results demonstrated that *GhirGDSL26* plays a critical role in cotton drought stress tolerance. Current findings enrich our knowledge of *GDSL* genes in cotton and provide theoretical guidance and excellent gene resources for improving drought tolerance in cotton.

**Supplementary Information:**

The online version contains supplementary material available at 10.1186/s12870-022-04001-0.

## Background

Increased levels of greenhouse gases in the atmosphere coupled with climate change are resulting in sporadic drought spells, which is a challenging issue in cotton production [1]. Cotton (*Gossypium hirsutum*. L), is an important industrial crop that provides 35% of the total fiber used globally. In recent years, drought and heat stress induced a 34% lint yield loss [2] as well as fiber quality [3–5]. Cotton is more sensitive to drought stress at the flowering and boll setting stage [6]. Therefore, improving cotton stress tolerance and breeding excellent stress-resistant varieties of cotton are of great significance to ensure high yield under hostile environments.

Plants cope with abiotic stresses using morpho-physiochemical and molecular adaptations [7]. Under water deficit conditions, plants close their stomatal apertures to reduce transpiration and maintain inner water potential to continue their normal growth [8]. Subsequently, plant leaves rolls, and wilt to regulate water loss [9]. Drought stress initially arrests root growth and then leaves and stems [10]. Meanwhile, a strong defensive system such as higher ion permeability, peroxide content, and antioxidant enzyme activities are activated [11]. Under drought conditions, cotton plants shows different morpho-physiological adaptations i.e., stomatal regulation, osmotic adjustment, thicker and smaller leaves, and enlarged tap roots [5, 12–15]. A significant amount of stress-associated genes is stimulated to cope with drought stress [7, 16].

GDSL esterase/lipases proteins (GELP), a subclass of lipid hydrolysis enzymes (lipolytic enzyme) with a conserved GDSL motif at the N-terminus are existed in all living organisms. It can be distinguished from other lipases with a special motif (GxSxG) near the center of their protein [17, 18]. GELPs have five consensus sequences (I-V) and four invariant important residues Ser, Gly, Asn, and His in four conserved blocks I, II, III, and V, respectively [17]. Due to their flexible active site, GDSL esterase/lipases have multifunctional properties such as broad substrate specificity, regiospecificity, and stereoselectivity [17, 19]. To date, the genome-wide identification of the GDSL gene family has been systematically investigated in various species, and a total of 105 *GDSL* genes were identified in *Arabidopsis thaliana* [19], 159 in tobacco [20], 114 in rice [21], 121 in *Brassica rapa* [22], 193 in *Dasypyrum villosum* [18], and 194 in soybean [23].

The *GELP77* regulates pollen wall characteristics in Arabidopsis and *GELP77*-deficient plants exhibited male sterility [24]. Similarly, *ZmMs30*, a novel type of GDSL lipase with diverged catalytic residues is essential for male fertility in maize, and loss of *ZmMs30* function induced defective anther cuticle, irregular foot layer of pollen exine, and complete male sterility [25]. In rice GDSL lipase *MHZ11* localized to the endoplasmic reticulum membrane and has acyl-hydrolyzing activity, reduces sterol levels to impair receptor-*OsCTR2* interactions and *OsCTR2* phosphorylation to stimulate ethylene signaling [26]. A GDSL lipase gene obtained from *G. hirsutum* was involved in ovule, seed, and fiber development [27]. The *OSP1* was involved in wax biosynthesis and stomatal cuticular ledge formation in Arabidopsis. *OSP1* mutation can result in a significant reduction in leaf wax synthesis and occlusion of stomata which leads to increased epidermal permeability, decreased transpiration rate, and enhanced drought tolerance [28]. Overexpression of *AtGDSL1* in *B.napus* can increase ROS and salicylic acid, reduces jasmonic acid levels, and enhances resistance of *Sclerotinia sclerotiorum* [29]. Triticeae *DvGELP53* negatively regulates resistance to barley stripe mosaic virus infection, and its knockdown can inhibit the long-distance movement of BSMV in the *Dasypyrum villosum* tissue [18]. A higher expression level of *SaGLIP8 in Sedum alfredii* Hance confers cadmium stress tolerance [30]. In soybean, overexpression of *GmGELP28* can induce drought and salt stress tolerance [23].

However, the GDSL gene family function under drought stress particularly in the cotton has not been reported yet. Multiple cotton genomes have been completed and published, which enables the identification of the GDSL family genes and their responses to drought stress [31–35]. In this study, we performed a genome-wide identification of GDSL in multiple cotton species and analyzed their phylogenetic relationship, chromosomal distributions, protein structure, and cis-element analysis. Under drought stress, we found that *GhirGDSL26* had an increased expression level through combined analysis of RNA-seq data and RT-qPCR. Meanwhile, *GhirGDSL26* was knocked out by virus-induced gene silencing in cotton and overexpressed in *Arabidopsis* (Col-0) to study underlying mechanisms involved in drought stress tolerance.

## Results

### Identification and phylogenetic analysis of the GDSL gene family in cotton

**Fig. 1:**
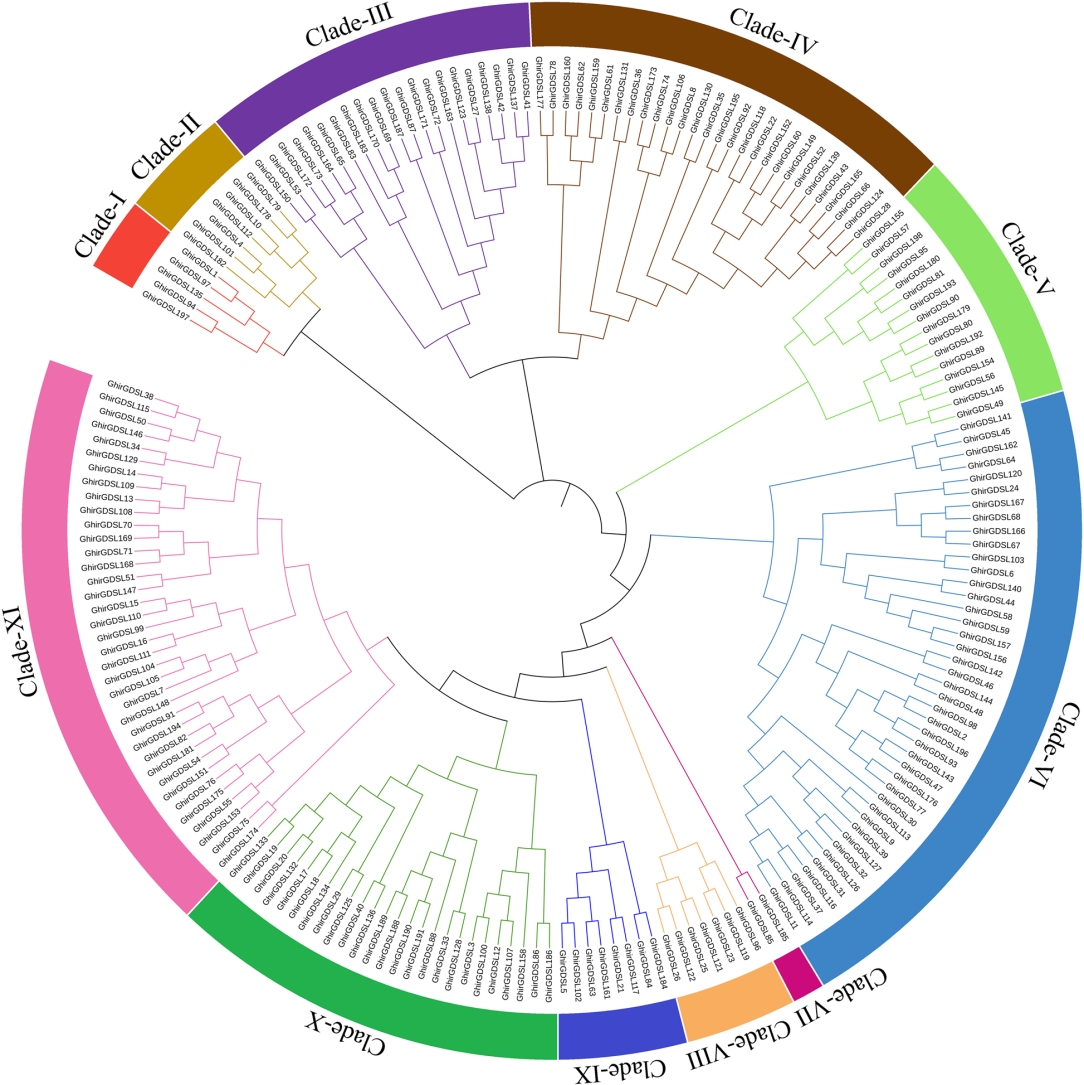
Phylogenetic relationship of GDSL gene family from *G. hirsutum*. The GDSL proteins were divided into eleven clades: Clade I to XI

A total of 103, 103, 99, 198, 203, 239, 249, and 215 *GDSL* genes were identified from eight cotton genomes of *G. herbaceum* (A_1_), *G. arboretum* (A_2_), *G. raimondii* (D_5_), *G. hirsutum* (AD_1_), *G. barbadense* (AD_2_), *G. tomentosum* (AD_3_), *G. mustelinum* (AD_4_), G. darwinii (AD_5_), respectively. These genes were designated *GheGDSL1*—*GheGDSL103*, *GarGDSL1—GarGDSL103, GraGDSL1—GarGDSL99, GhirGDSL1—GhirGDSL198, GbarGDSL1—GbarGDSL239, GtomGDSL1—GbarGDSL 239, GmusGDSL1—GmusGDSL249, and GdarGDSL1—GdarGDSL215* according to their chromosomal locations (Table S1). The protein isoelectric point and molecular weight of all GDSL members were examined (Table S1). To assess the evolutionary relationships of GDSL members, a phylogenetic tree was constructed based on the full-length sequences of 198 GDSL proteins from *G. hirsutum* (Fig. 1). All *GhirGDSLs* were divided into eleven clades (I-XI), and *GhirGDSLs* were unevenly distributed in different clades. A total of 5, 7, 21, 28, 16, 42, 2, 7, 8, 25, and 37 *GhirGDSLs* were found in Clade I through Clade XI, respectively. Clade VI was the largest clade with 42 members followed by Clade IV (28 members) and Clade X (25 members). The smallest clade was Clade VII containing only two members.

### Chromosomal allocation, gene structure, and motif identification

**Fig. 2:**
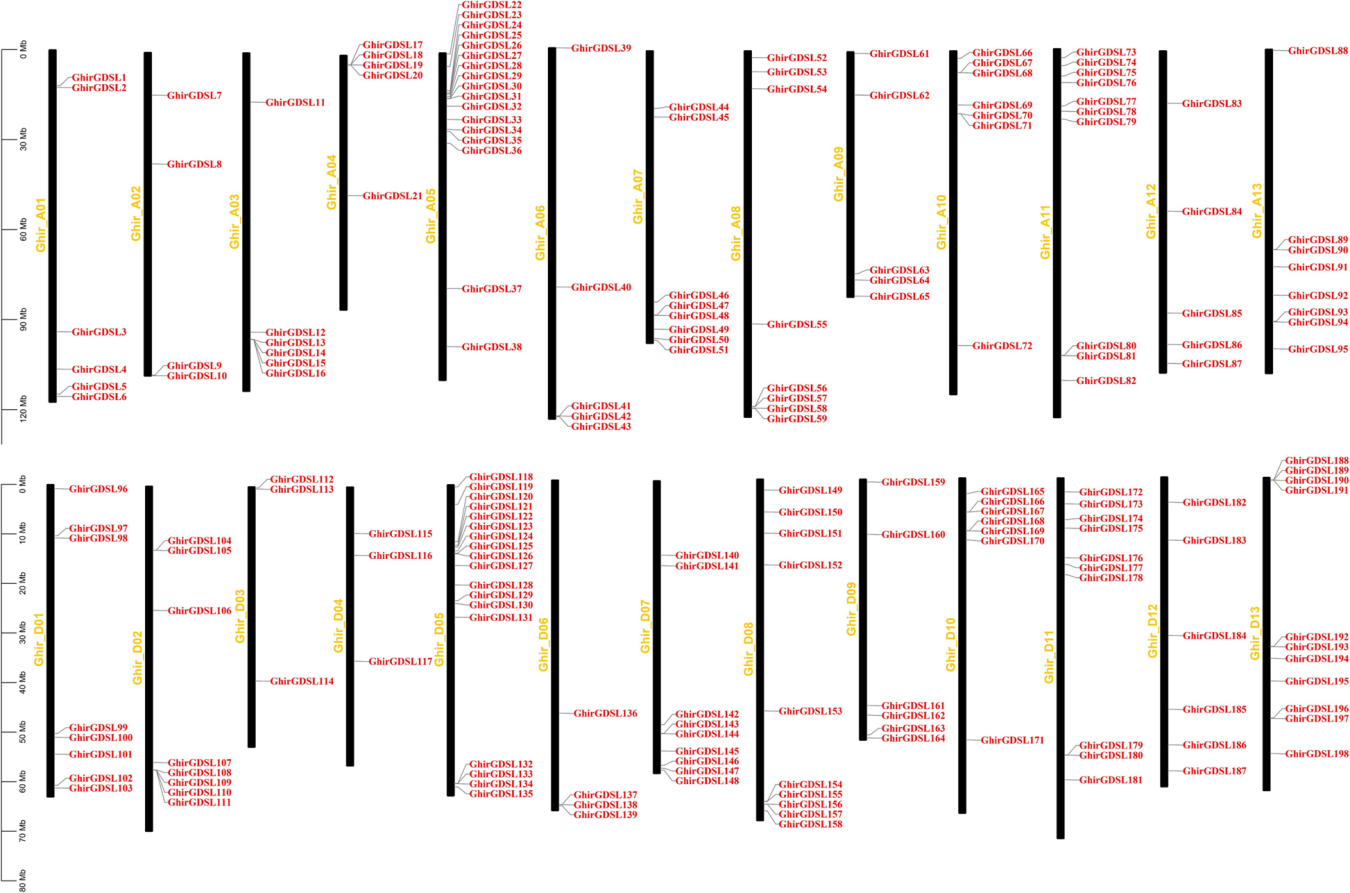
Chromosomal locations of *GDSLs* in *G. hirsutum*. The chromosome numbers were marked left of each chromosome in blue. The *GDSL* genes' names are marked in red. The bar located on the left side showed the size of the chromosome in megabases

In *G. hirsutum*, 198 *GDSL* genes are universally and unevenly distributed to all 26 chromosomes. The number of *GDSL* genes distributed on different chromosomes was different. The more gene loci were observed on chromosomes D05 and A05 with 18 and 17 genes respectively, while the less gene loci were observed on chromosomes D03 and D04 with 3 genes each (Fig. 2). *GhirGDSL* genes were mainly located on chromosome ends, and only a few genes were found in the middle of the chromosomes A04, A12, and D13. *GhirGDSL*s were commonly presented as clusters, such as *GhirGDSL22-36* on chromosome A05 and *GhirGDSL118-131* on chromosome D05.

**Fig. 3:**
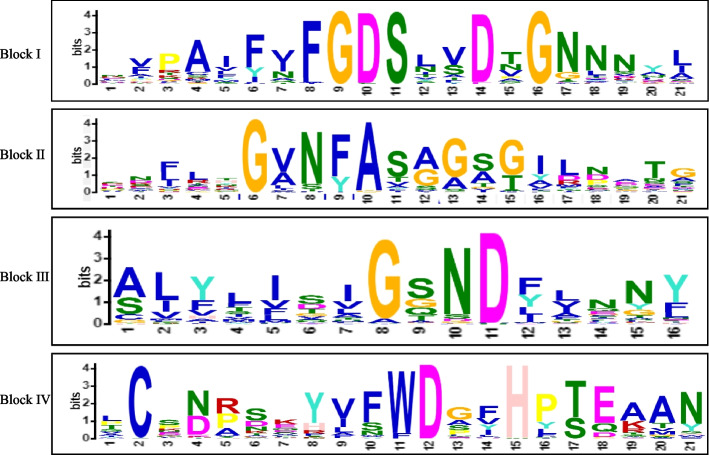
Motif logos of four conservative blocks were detected in GhirGDSL proteins. Conserved amino acid residues Ser-Gly-Asn-His were marked by red triangles

The exon numbers, exon length, intron numbers, and intron length of *GhirGDSL* genes were calculated in this study (Table S2). Intron numbers among *GhirGDSL* varied from 1 to 12 (Table S2). All genes possessed one intron, and the highest numbers of introns were present in the *GhirGDSL39* and *GhirGDSL100* with 12 introns each. Most of *GhirGDSLs* were found to have four introns (135 out of 198, 68.2%), followed by *GhirGDSLs* having three, two, and five introns, which account for 11.1% (22), 9.1% (18), and 6.6% (13), respectively. The *GhirGDSLs* members with one, six, and seven introns were rare. A total of 10 conserved motifs were detected in the GhirGDSLs family. Motifs 4, 5, 9, and 1 were present in almost GDSL proteins, which represent conservative blocks I, II, III, and V of the GhirGDSLs family, respectively (Fig. 3).

### *Cis*-Acting regulatory element identification and subcellular localization prediction

**Fig. 4:**
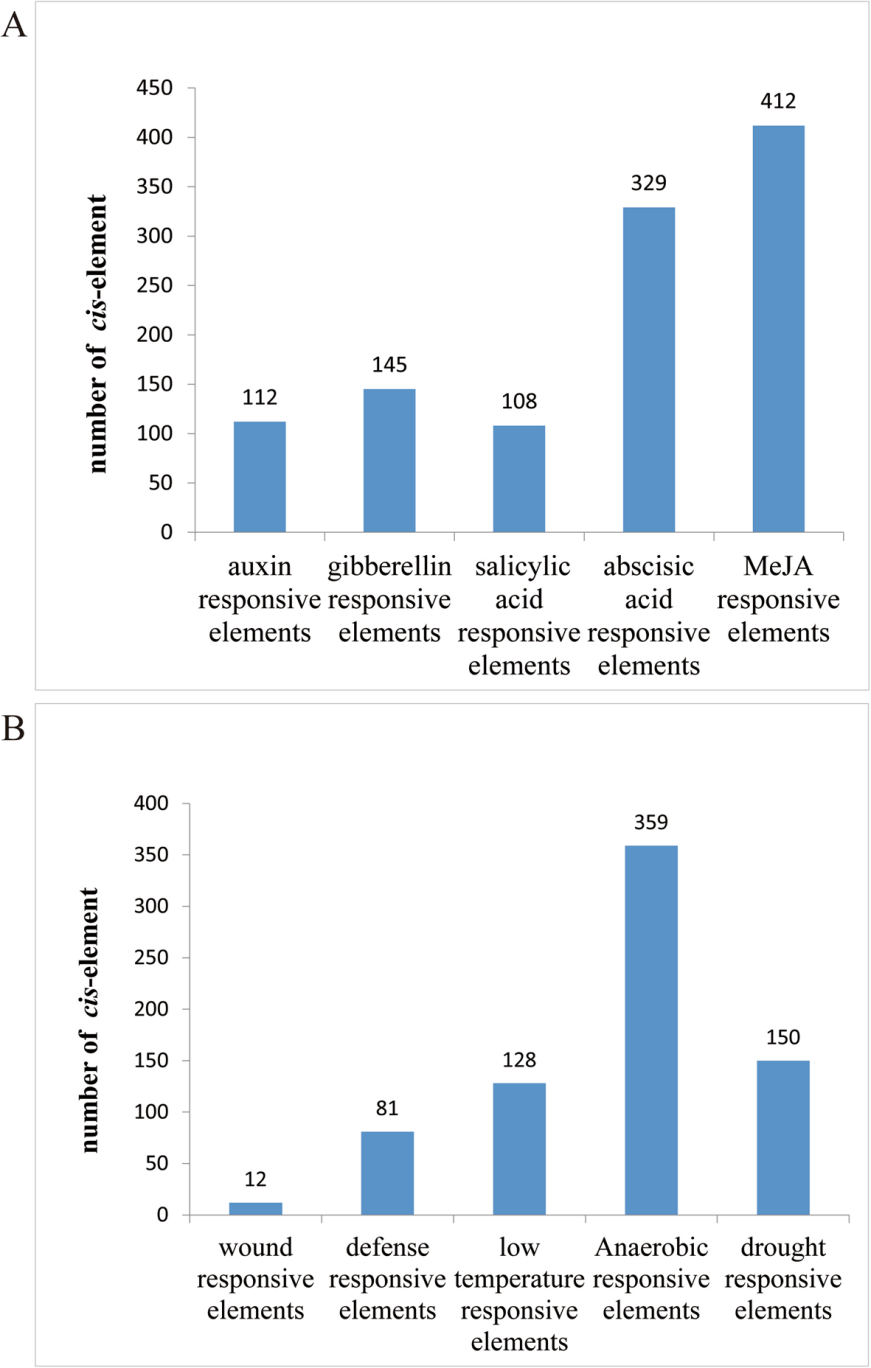
The *cis*-elements analysis of the *GhirGDSLs* promoter sequence. **A** The classification and number of hormone-responsive elements. **B** The classification and number of environmental stress-responsive elements

The 2000 bp upstream sequences from the translational start site were downloaded from CottonGen (https://www.cottongen.org/) and submitted to PlantCARE to analyze the associated *cis*-regulatory elements in the promoter region. In the promoter regions of all 198 *GhirGDSL* genes, there were abundant o*cis*-elements associated with plant hormones and environmental stress besides light-responsive elements. The light-responsive elements are the most common (2424), followed by hormone-related *cis*-elements (1006) and environmental stress elements (730). In the hormone-related *cis*-elements, auxin, gibberellin, salicylic acid (SA), abscisic acid (ABA) and MeJA responsive elements were 112, 145, 108, 329, and 412, respectively (Fig. 4A). In the environmental stress elements, 12 were wound responsiveness, 81 were defense and stress responsiveness, 128 were low-temperature responsiveness, 150 were drought induction, and 359 were anaerobic induction elements (Fig. 4B). The enrichments of SA responsive elements, ABA-responsive elements, low-temperature responsive elements and drought-responsive elements in the promoters of *GhirGDSL* genes indicated that numerous *GhirGDSL* genes may be involved in plant responses to cold and drought stress.

An online tool WoLF PSORT was employed to determine the possible Subcellular localization of the proteins encoded by *GDSL* genes. The results showed that GDSL proteins are widely distributed in the nucleus, cytoplasm, chloroplast, vacuole, mitochondria, Golgi apparatus, and so on.

### Expression analysis of *GhirGDSL* genes by RNA-Seq and RT-qPCR

**Fig. 5:**
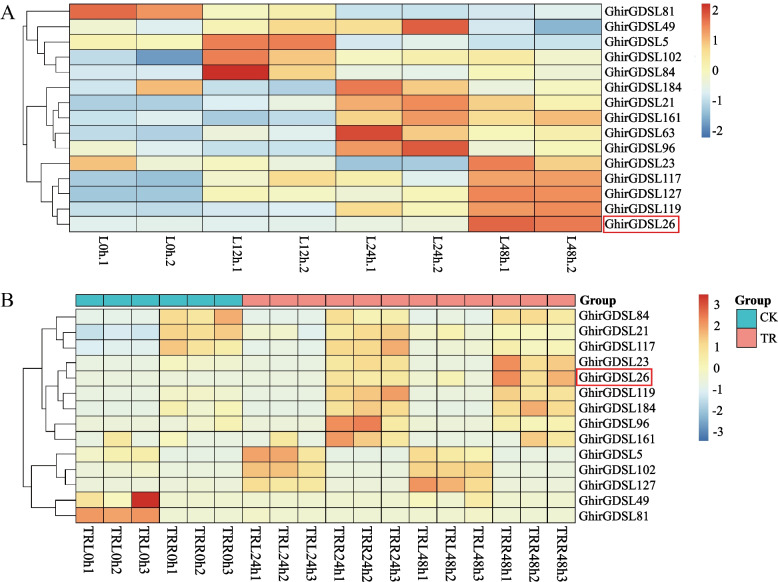
Expression analysis of *GhirGDSL* genes in *G. hirsutum* under drought stress. **A** RT-qPCR analysis of the selected *GhirGDSL* genes under drought stress conditions, imposed by adding 16% of PEG-6000. **B** The RNA-Seq expression profiles of selected fifteen *GhirGDSL* genes of *G. hirsutum* under drought stress. Levels of gene expression were depicted in different colors on the scale. Red color represents high expression and blue color represents low expression. The red box indicates the key candidate gene. CK represents “control check”, and TR represents “treatment”. Three biological replications and three technical replications for the RT-qPCR experiment were taken

The sequence alignments between *Gossypium hirsutum* and *Arabidopsis GDSL* genes, we found that there were 15 *GhirGDSL* genes that were highly similar, and the expression patterns of these 15 genes under drought stress were analyzed using the *G. hirsutum* Mg85 transcriptome data [36] (NCBI accession: PRJNA663204). However, out of the 15 identified *GhirGDSL* genes, 14 genes showed different expression patterns in CK and treatment group at the same stage. Among these fourteen genes in *G. hirsutum*, eleven genes had significantly up-regulated expressions and only three genes were found to be down-regulated. Subsequently, we selected the fourteen differentially expressed genes for further RT-qPCR validation (Fig. 5A). The results of RT-qPCR showed similar expression patterns as the transcriptome data (Fig. 5B). Based on the transcriptome data and RT-qPCR results, we selected the *GhirGDSL26,* one of the highly up-regulated gene under drought stress for further validation.

### Heterologous expression of *GhirGDSL26* in *A. thaliana* confers enhanced drought tolerance

**Fig. 6:**
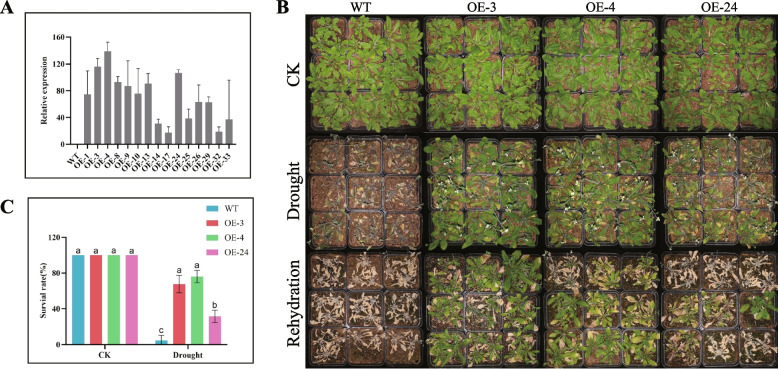
Phenotype identification of *GhirGDSL26* overexpressed lines and WT (Col-0) under natural drought stress. **A** Relative expression levels of transgenic lines by RT-qPCR. *ATactin* was used as an internal control. **B** Phenotypic observation of the overexpressed lines and WT under natural drought treatment and after rehydration treatment. **C** The survival rates of overexpressed lines and WT. Three biological replications and three technical replications for each experiment, Student’s *t*-test were used to determine the mean comparison with ± SD at *P* < 0.05. Means with different lowercase letters show a significant difference

To identify the function of the target gene, we over-expressed the *GhirGDSL26* gene from *G. hirsutum* into the *A. thaliana* (Col-0) through the floral-dip method. A total of 10 independent transgenic lines were generated and cultivated in a selective medium containing kanamycin. Subsequently, the expression of *GhirGDSL26* in the T2 transgenic lines was examined using RT-qPCR. The levels of *GhirGDSL26* expression were significantly up-regulated in all transgenic lines (Fig. 6A). Based on these result, three transgenic lines (OE-3, OE-4, and OE-24) with higher expression of *GhirGDSL26* were selected for generating T3.

To evaluate drought tolerance of transgenic lines, the OE-3, OE-4, OE-24, and wild type (WT) (Col-0) were tested drought conditions, (naturally induced drought stress and 200 mM mannitol treatments). naturally induced drought stress, the three transgenic lines, and wild types were sowed in the nutrition pots for two weeks under normal conditions. To induce the natural drought stress we stopped watering plants for two weeks. There were no significant differences in the phenotype of three overexpressed lines and WT under the CK conditions. All the plants observed at CK showed normal growth. But, the transgenic lines showed a more tolerant phenotype with less wilted leaves compared with the WT after two weeks through natural drought treatment (Fig. 6B). After rehydration treatment, the survival of wild-type plants was limited, but the overexpressed *GhirGDSL26* plants were able to recover growth. The survival rates of the OE-3, OE-4, and OE-24 transgenic lines were 67.6%, 75.9%, and 31.48%, respectively, whereas the WT plants exhibited a lower survival rate (4.6%) (Fig. 6C).

**Fig. 7:**
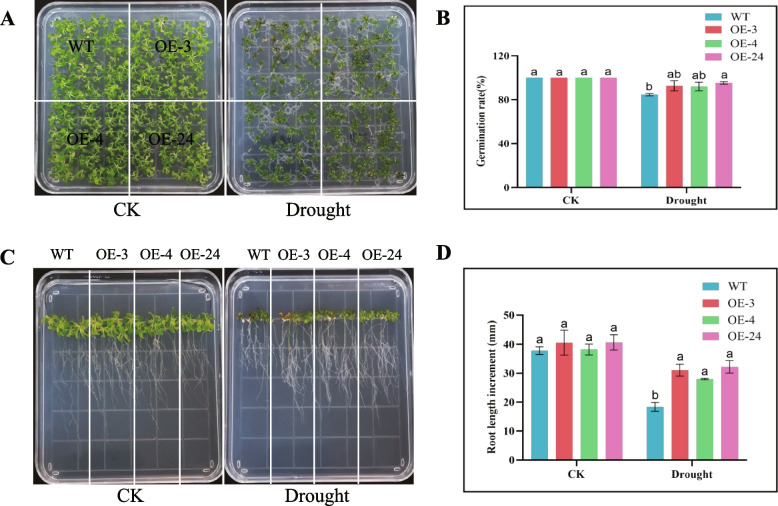
Determination of germination rate and root length of the *GhirGDSL26* overexpressed lines and WT (Col-0) under drought stress. **A** The germination rate of the overexpressed lines and WT under the CK and drought stress (200 mM mannitol). **B** Germination rates under control and drought stress conditions. **C** Root length of the overexpressed lines and WT under the CK and drought stress (200 mM mannitol). **D** Root length statistic chart. Three biological replications and three technical replications for each experiment were taken, Student’s *t*-test was used to determine the mean comparison with ± SD at *P* < 0.05. Means with different lowercase letters show a significant difference

Under the mannitol indued drought, 30 sterile seeds of WT (Col-0), OE3, OE4 and OE24 were planted in the plates with 1/2 MS medium containing 200 mM mannitol. The germination rates of three transgenic lines and WT seeds were approximately 100% under normal conditions. However, the germination rates of WT were decreased by 84.7% under drought stress compared with OE-3, OE-4, and OE-24 lines, 92.7%, 92.0%, and 95.3%, respectively. A significant difference between the germination rate of the overexpressed lines and WT was noticed (Fig. 7A, B). Root lengths of the three transgenic lines and WT were similar in the CK, whereas the root lengths of the OE-3, OE-4, and OE-24 lines were significantly longer as compared to wild-type plants under drought stress (Fig. 7C, D).

### Cell damage in transgenic plants under drought stress conditions

**Fig. 8:**
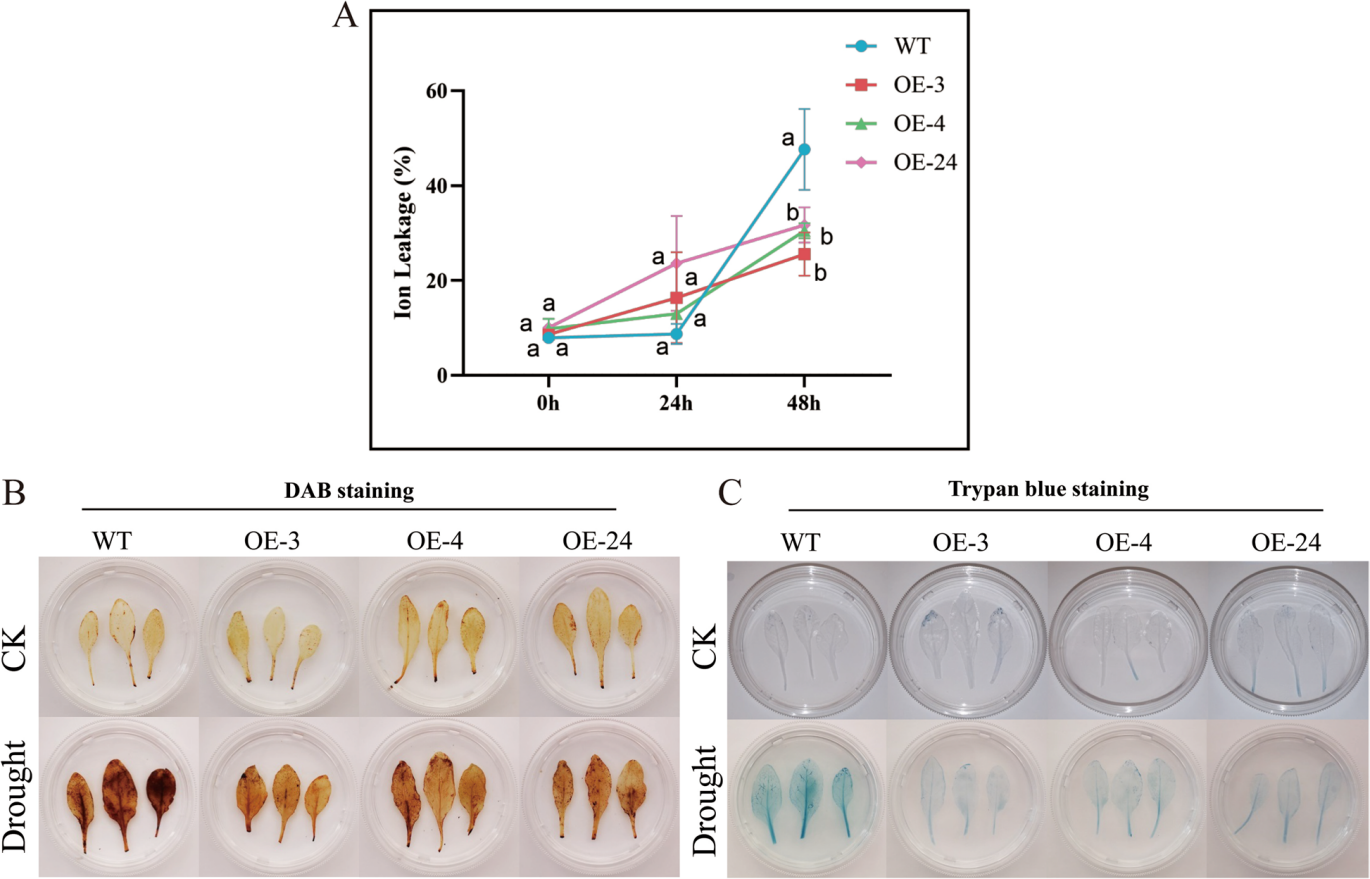
Evaluation of cell damage of the overexpressed lines and WT (Col-0) under drought stress. **A** Estimation of Ion leakage (**B**) 3,3’-Diaminobenzidine (DAB) staining. **C** Trypan blue staining. Three biological replications and three technical replications for each experiment were taken, Student’s *t*-test was used to determine the mean comparison with ± SD at *P* < 0.05. Means with different lowercase letters show significant difference

In WT (Col-0) plants, the level of stress-induced ion leakage was substantially higher than the transgenic lines after 48 h of drought treatment. Current results indicate that the WT plants had a higher leaf cell damage (Fig. 8A). Leaves of transgenic and WT plants were stained with DAB to measure H_2_O_2_ levels. Under drought stress, the color of the stained WT leaves was more darker as compared to the three overexpressed lines. Results suggests that the transgenic lines had a lower H_2_O_2_ content as compared to the wild type plants (Fig. 8B). Similarly, trypan blue staining indicated that the cell damage in wildtype leaves was much higher as compared to the overexpressed lines under drought treatment (Fig. 8C).

### Expression patterns of stress-responsive genes in response to drought stress

**Fig. 9:**
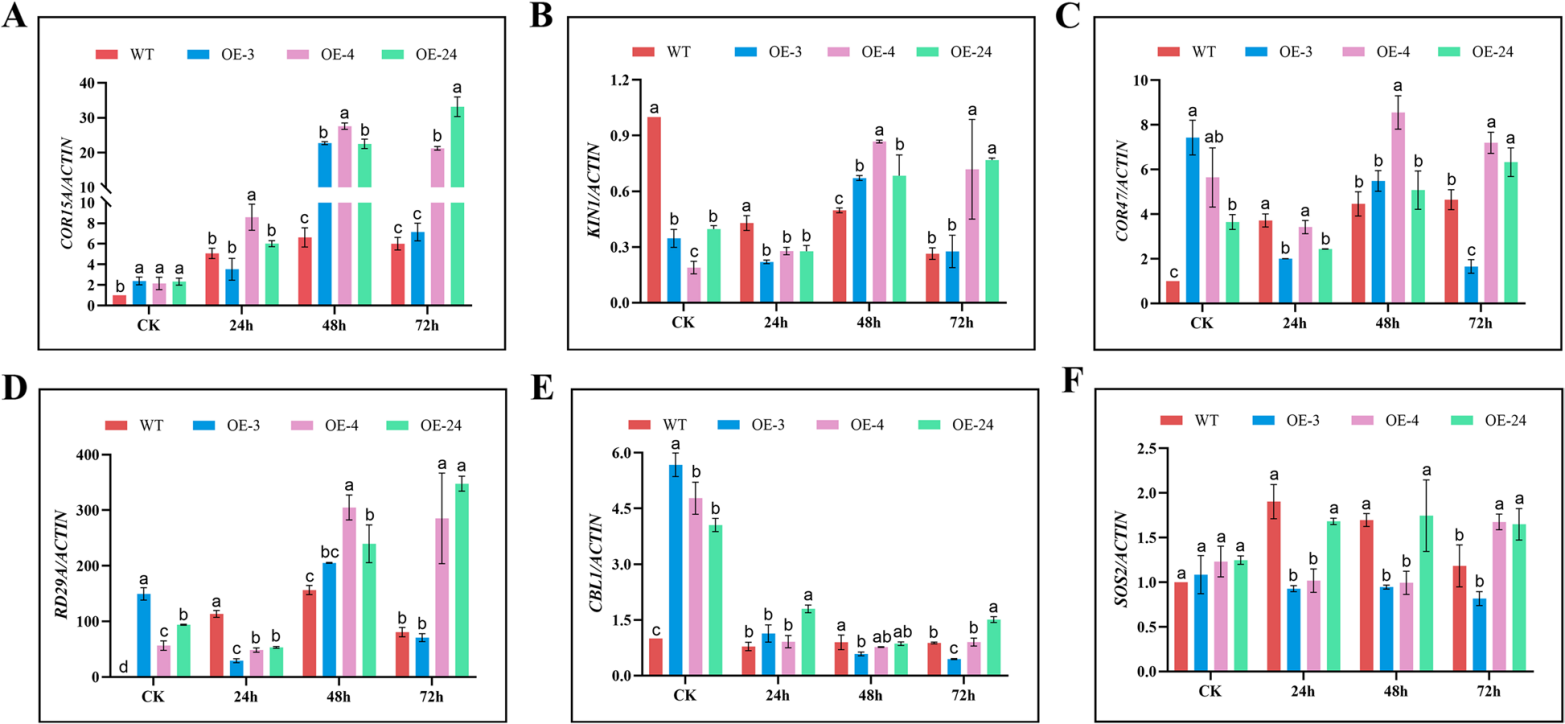
Expression patterns of stress-responsive genes in *GhirGDSL26* overexpressed lines and WT (Col-0) under drought stress. **A** Expression patterns of *COR15* responsive gene. **B** Expression patterns of *KIN1* responsive gene. **C** Expression patterns of *COR*47 responsive gene. **D** Expression patterns of *RD29* responsive gene. **E** Expression patterns of *CBL1* responsive gene. **F** Expression patterns of *SOS2* responsive gene. Three biological replications and three technical replications for each experiment were taken, Student’s *t*-test was used to determine the mean comparison with ± SD at *P* < 0.05. Means with different lowercase letters show a significant difference

RT-qPCR was utilized to assess the expression of six stress-responsive genes in WT (Col-0) and *GhirGDSL* transgenic lines under drought stress. Under the controlled conditions, No difference in the expression of six stress-responsive genes(*COR15A*, *COR 47*, *RD29A*, *CBL1* and *SOS2*) were found between three transgenic lines and wild types (Fig. 9A-F) Under drought stress, *COR15A* gene was up-regulated in both overexpressed plants and WT plants at early stages of drought treatment (24 h), and its expression significantly increased with drought prolongation and reached the peak at 48 h. But the expression of *COR15A* gene in the three transgenic lines were obviously higher than that in the WT plants (Fig. 9A). As drought stress prolongs, the expression of *KIN1* gene in the transgenic plants increased gradually and subsequently to a stable level, but its expression in the WT plants was down-regulated under drought stress, and kept at a low and stable level in the entire duration of the drought stress (Fig. 9B). The expression trends of *COR47*, *RD29A* and *SOS2* were similar with the *COR15*A expression (Fig. 9C, D and F). However, the *CBL1* gene in the overexpressed lines was sharply down-regulated under drought stress (Fig. 9E). Generally, most of the stress-responsive genes were more significantly induced in transgenic lines than that in wild type plants under drought stimulus, suggesting that heterologous expression of *GhirGDSL*26 causes a series of stress-responsive genes altering and enables a drought tolerance potential in transgenic plants.

### Knockdown of *GhirGDSL26* in cotton via Virus-induced gene silencing (VIGS)

**Fig. 10:**
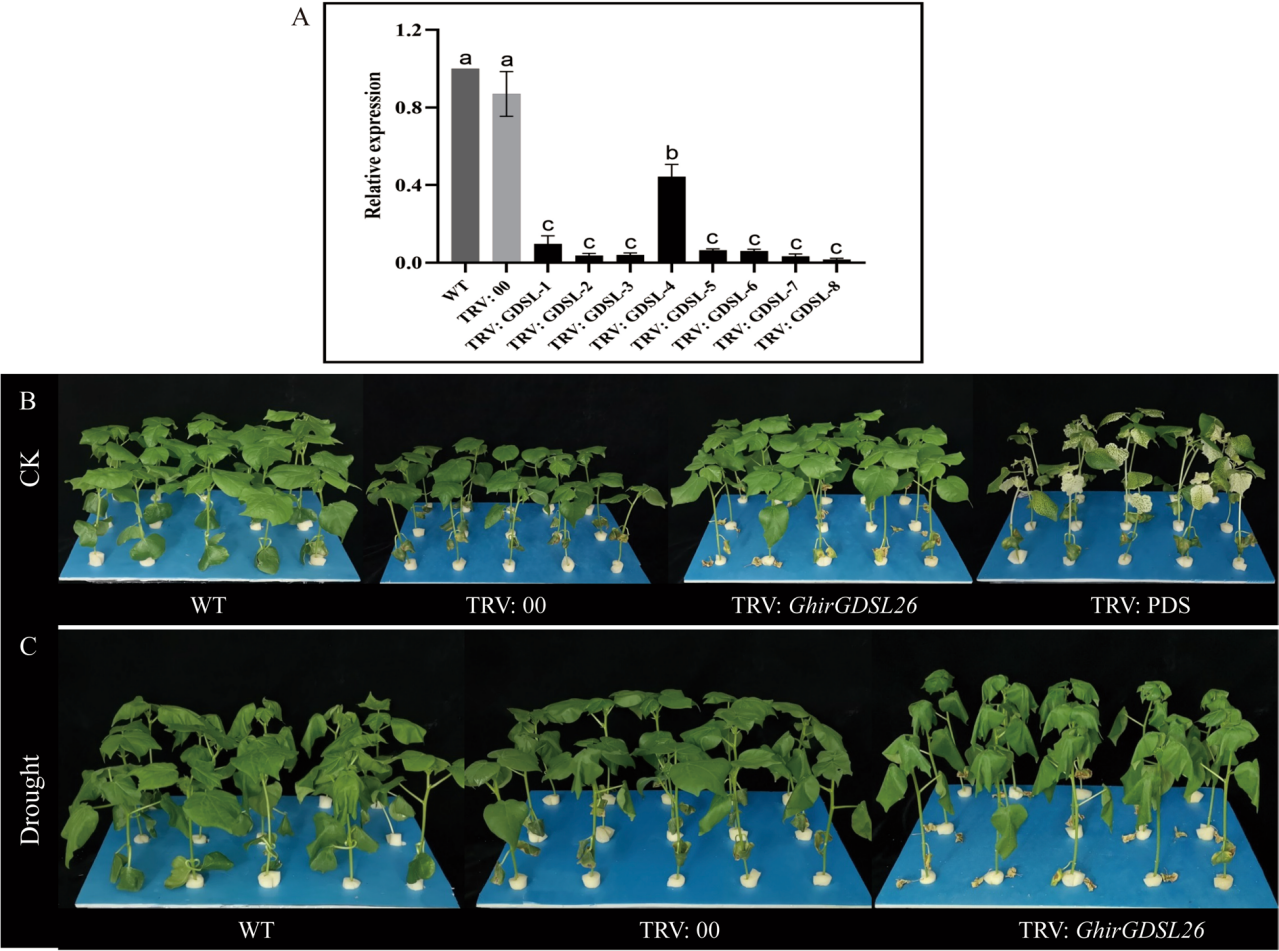
Phenotypic variations in the VIGS-plants and WT under drought stress. **A** VIGS interference detection of *GhirGDSL26* gene expression level. **B** Phenotype representation of WT, empty vector (TRV:00), PDS albino phenotype (TRV:PDS), and *GhirGDSL26* under the CK conditions. **C** Phenotypic representation of WT, empty vector (TRV:00), and *GhirGDSL26* under drought stress

VIGS was used to knock down *GhirGDSL26* in *G. hirsutum*. After the injection of PDS to three-leaf cotton seedlings, the albino phenotype appeared approximately 8 days later and remained stable for a month. This confirmed that gene silencing was successful and the silencing efficiency was stable. The expressions of *GhirGDSL26* in WT, TRV:00, and TRV: *GhirGDSL26* plants were measured using RT-qPCR. No significant differences in the gene expression between WT and TRV:00 plants were recorded, whereas the gene expression in the TRV: *GhirGDSL26* plants were significantly lower than WT and TRV:00. This indicates the *GhirGDSL26* gene was successfully silenced in the cotton (Fig. 10A). Under normal conditions, no significant variations were observed in plant phenotype between the VIGS plants and the WT (Fig. 10B). Under drought stress conditions, the VIGS plants growth was significantly retarded with increased leaf wilting, but the WT and TRV:00 plants just exhibited slight water-soaking in the leaves (Fig. 10C). Overall, we can assume that silencing of *GhirGDSL26* in cotton cause more phenotypic damage under drought stress as compared to WT and TRV:00 plants.

**Fig. 11:**
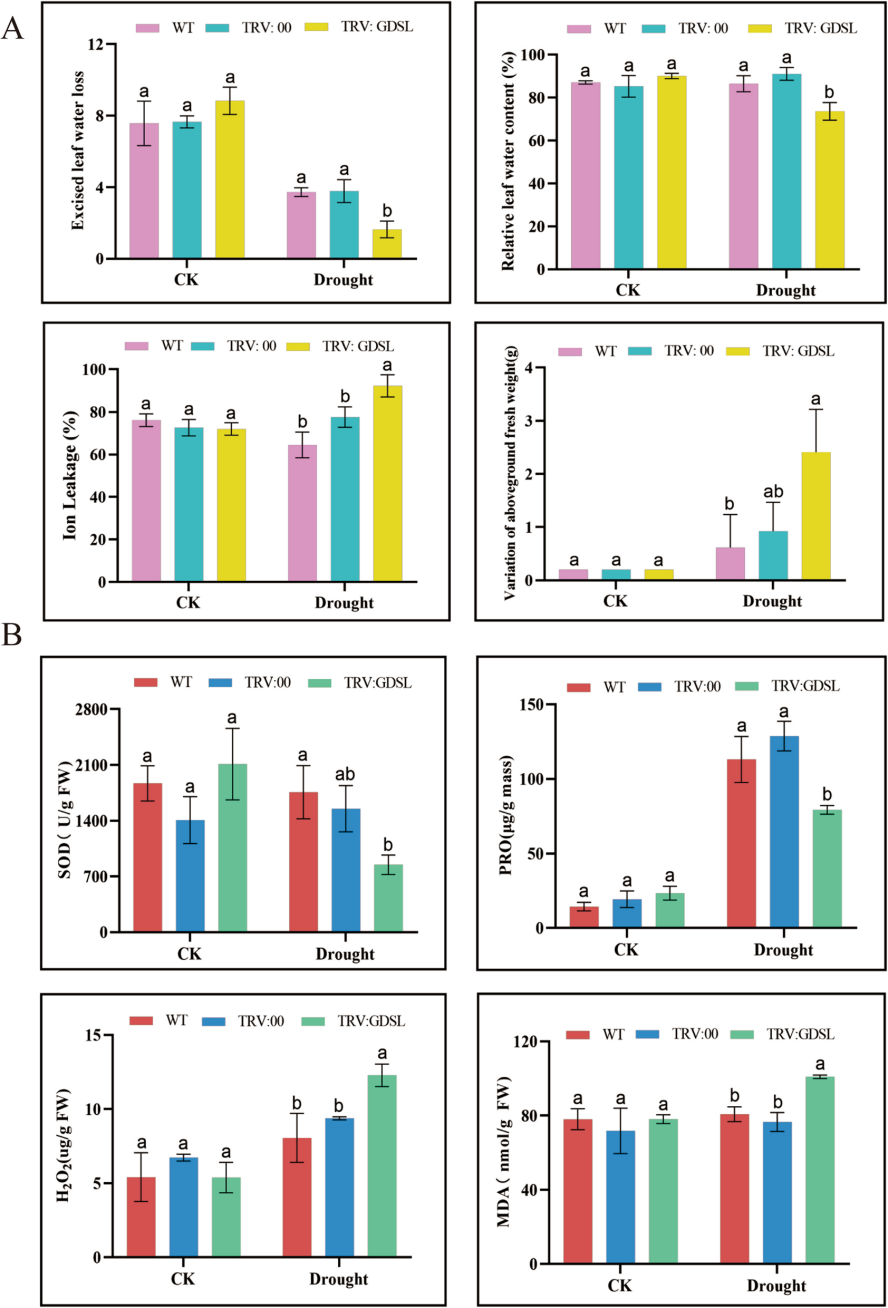
Physio-biochemical traits in VGIS-plants and WT under drought stress. **A** Determination of ELWL (Excised leaf water loss), RLWC (Relative leaf water content), IL (Ion leakage), and VAFW (Variations in aboveground fresh weight). **B** Determination of superoxide dismutase (SOD) activities, proline (PRO) content, hydrogen peroxide (H_2_O_2_) content, and MDA content. Three biological replications and three technical replications for each experiment were taken, Student’s *t*-test was used to determine the mean comparison with ± SD at *P* < 0.05. Means with different lowercase letters show a significant difference

Compared with the WT and TRV:00 plants, the TRV-*GhirGDSL26* plants had a higher VAFW (Variations in aboveground fresh weight), IL (Ion leakage), MDA, and H_2_O_2_ contents with reduced ELWL (Excised leaf water loss), RLWC (Relative leaf water content), PRO and SOD (Fig. 11). Taken together, these findings demonstrated that *GhirGDSL26* plays a positive role in mediating cotton drought tolerance and silencing*GhirGDSL26* makes plants more vulnerable to damage due to drought stress.

## Discussion

Cotton plants are considered more drought-tolerant than other crops. However, prolonged water deficit conditions can significantly hinder cotton growth resulting in yield loss. Wild cotton germplasm resources under long-term natural selection can display more diversity and obvious superiorities under harsh environments over currently cultivated varieties [37, 38]. GDSL esterase/lipase proteins have been identified in many plant species and induce tolerance to abiotic stresses [39]. The understanding of the biological function of *GDSL* genes and their associated molecular mechanisms under harsh environments will help in developing new cotton cultivars with high tolerance to harsh environmental conditions. However, due to the large and complex genomes of cotton, no data is reported on the function of *GDSL* genes so far [27, 40].

In this study, a total of 103, 103, and 99 *GDSL* genes were found in the *G. herbaceum* (A_1_), *G. arboretum* (A_2_), and *G. raimondii* (D_5_)*,* respectively, which are similar to *Arabidopsis* (105) [19] and rice (114) [21]. In the allotetraploid cotton, 198, 203, 239, 249, and 215 were identified from *G. hirsutum* (AD_1_), *G. barbadense* (AD_2_), *G. tomentosum* (AD_3_), *G. mustelinum* (AD_4_) and *G. darwinii* (AD_5_), respectively. Moreover, *GDSL* genes are commonly existed in cluster form in most chromosomes as reported in soybean [23] and *Dasypyrum villosum* [18]. This phenomenon may be caused by gene duplication during evolution. Phylogenetic analysis displayed that each clade included *GDSL* members from cotton, cacao, Arabidopsis, and rice, indicating GDSL family originated and diversified before the divergence of these species (Fig. S1). Although, clades I and VII comprised fewer members but were conserved across all species. This further explained that *GDSL* genes are the main drivers for regulating biological processes. Moreover, the GDSL proteins are extensively localized in the cytoplasm, endoplasmic reticulum, dictyosome, mitochondrion, cytoplasm, vacuole, and extracellular and are responsible for complex and diverse functions performed by the GDSL gene family.

Gene structure is one of the representative traces of gene family evolution [41]. A striking feature of plant *GDSL* genes is their structure of five exons and four introns [23]. In this study, all *GhirGDSL* genes carried one to twelve introns and two to thirteen exons. Phylogenetic analysis divided all *GDSL* genes into eleven clades which showed different intron and exon numbers among clades. These differences were the result of *GhirGDSL* genes-induced changes in intron and exon during evolution. In the present study, up to 68.2% of *GhirGDSL* genes contained five exons and four introns, which are consistent with the results reported in other plants [19, 23]. Besides, the transcription levels of genes can be influenced by exon/intron numbers. For instance, genes with fewer introns/exons can be faster expressed [42, 43]. In addition, the majority of the *GhirGDSL* genes had conserved motifs (black I, II, III, and V), which further confirmed that *GhirGDSL* genes were conserved during evolution.

The transcriptional activation level in eukaryotes is regulated by upstream *cis*-acting elements in the regulation of gene expression, which are key players in plant response to stress [44]. ABREs are a class of elements capable of binding to strongly conserved abscisic acid (ABA)-dependent transcription factors. These are presented in the promoter region of many stress-resistant genes and can regulate related gene expression under harsh environments [45]. In the current study, we observed that the promoters of *GhirGDSL* genes contains conservative elements, e.g., TATA-box and CAAT-box, and moreover, there were other *cis*-elements, such as light response elements G-box, LAMP-element, ABA response elements ABRE, and MYC, low-temperature response elements LTR, and SA related TCA-elements. Enrichments of these *cis*-acting elements suggest that *GDSL* genes may be involved in different biotic and abiotic stress responses.

GDSL gene family members are prerequisite for plant growth regulation, secondary metabolites synthesis, and adversity adaption [18, 39, 46]. In this study, gene expression was changed, under drought conditions revealing that the *GhirGDSL* gene induced drought stress tolerance in cotton. The expression patterns of all the 15 *GDSL* genes we selected were observed under drought stress. *GDSL26*, one of the 15 candidate genes, had a significant expression in both transcriptome and RT-qPCR data.

Heterologous expression of genes to validate their functions in response to biotic and abiotic stresses in Araidpopsis has been a best alterbative so far. Heterologous expression of *the GDSL* gene in *Arabidopsis* has proved drought tolerance. Heterologous expression of *Arabidopsis GDSL1* enhances tolerance against *Sclerotinia sclerotiotum* infection in rapeseed [29]. Overexpression of *GmGELP28* from soybean in Arabidopsis can enhance drought and salt stresses tolerance [23]. In this study, *GhirGDSL26*, a new drought-resistant candidate gene was isolated from the *G. hirsutum*-Mg85, semi-wild cotton that has the potential to enhance drought and salt stress tolerance [36]. Under drought stress, the *GhirGDSL26* over expressed *Arabidopsis* plants resulted in higher seed germination and survival rates over WT plants, suggesting that the *GhirGDSL26* gene has the potential to induce drought tolerance in cotton seedlings.

Plants being exposed to severe stress can result in a highly disintegrated cell membrane leading to an increased passive efflux of ions from cytosol to the outside [47]. In this study, *GhirGDSL26* transgenic lines had a lower ion leakage under drought stress, suggesting that *GhirGDSL26* transgenic plants had reduced membrane damage and improved drought tolerance. MDA is a substance produced by membrane lipids and can be used as a stress indicator to evaluate the degree of plasma membrane damage and the ability of plants to cope with drought stress [48]. SOD and CAT are the major antioxidant enzymes for the scavenging of O_2_^−^ and H_2_O_2_, and their activities are generally increased to maintain ROS homeostasis under stress conditions [49]. Free proline in a plant can stabilize proteins and cell structures under unfavorable conditions [50]. In this study, higher MDA and H_2_O_2_ contents and lower SOD activity and proline contents were noticed in the *GhirGDSL26*-silenced cotton seedlings compared with WT cotton plants. This indicated that *GhirGDSL26* is involved in drought-induced antioxidant defense systems and maintained cell homeostasis.

In this study, expressing the *GDSL* gene significantly increased induction levels of the stress-responsive genes. *COR15* can stabilize leaf cells through folding and binding to chloroplast membranes. Its accumulation is important for the acclimation of cold stress in Arabidopsis [51]. In the *GhirGDSL26* transgenic Arabidopsis, the expression of *COR15* was at its peak when exposed for 48 h of drought stress duration (Fig. 9A, B). While *COR47* was activated and the expression was at a peak at 48 h (Fig. 9C), which is moderately induced by ABA [52]. *RD29* could be activated by the MAPKK pathway [53] and promoted the expression of *EsWAX1* resulting in a higher accumulation of cuticular wax under drought stress in Arabidopsis [54]. In this study, the *GhirGDSL26* was over-expressed in Arabidopsis, and *RD29* has highly expressed at 72 h of stress duration (Fig. 9D), explaining that cuticular wax synthesis was not the first line but played a key role in response to drought stress. *CBL1* was a unique group of calcium sensors in plants that can positively regulate drought/salt responses, but mutant plants displayed less tolerance to drought stress [55, 56]. In the *GhirGDSL26* transgenic Arabidopsis, the expression of *CBL1* was suppressed which indicates that there is an antagonism between *CBL1* and *GDSL* to deal with drought stress in this study (Fig. 9E). *CBL1* suppression can increase vapor pressure difference in leaf-to-air to close the stomata to reduce excessive water loss [57]. *SOS2*, a salt overly sensitive signaling pathway component, modulates the vacuolar V-ATPase and regulates the Na^+^/H^+^ and the Ca^2+^/H^+^ exchange at the vacuolar membrane under drought stress [58, 59]. However, in this study, *SOS2* remained inactivated when *GhirGDSL26* was overexpressed under drought stress (Fig. 9F).

**Fig. 12:**
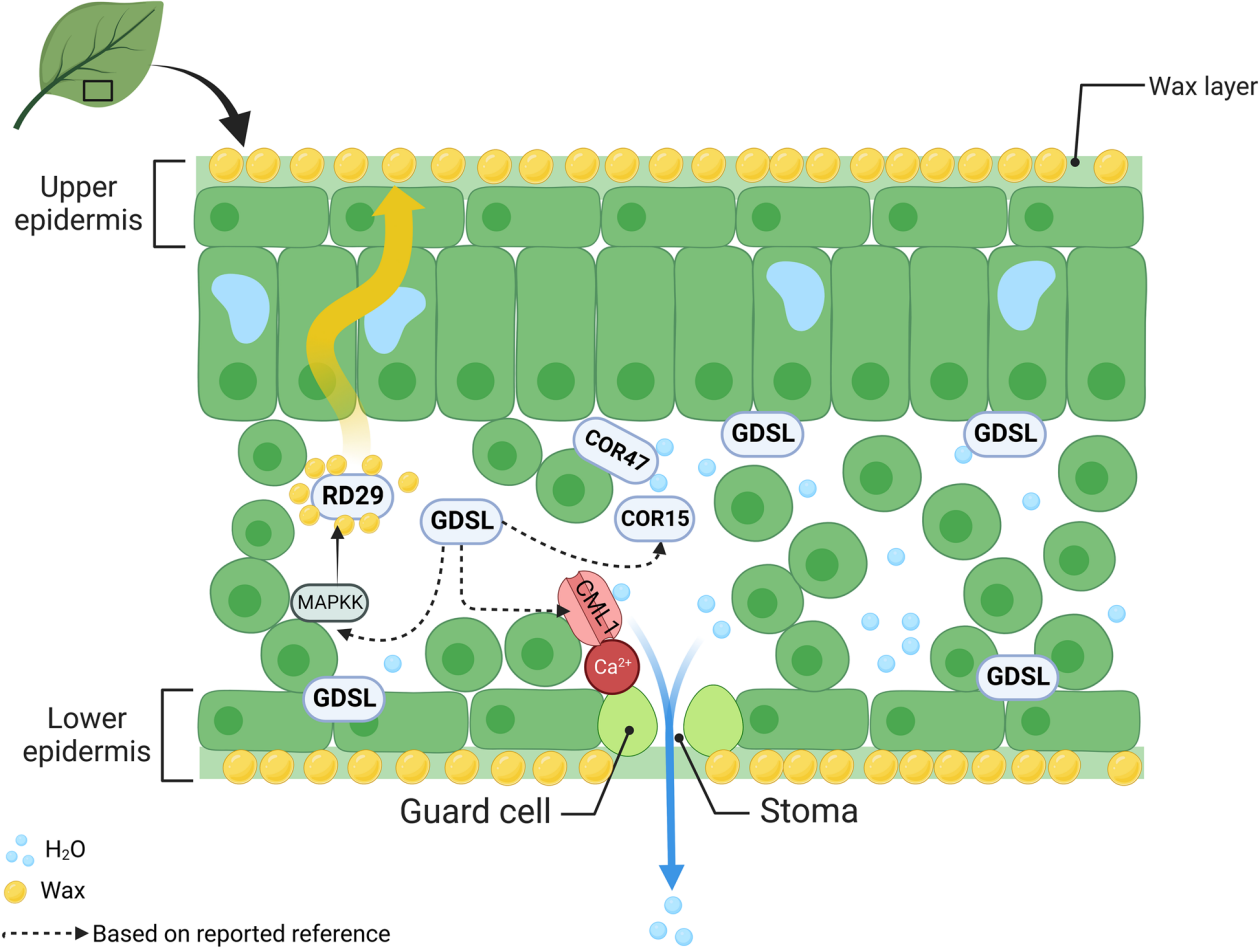
Schematic diagram of *GhirGDSL26* drought resistance mechanism

Our findings can offer a potential regulatory network of *GDSL*-induced drought tolerance in cotton seedlings (Fig. 12). *GDSL26* is widely distributed in lipids that respond to osmotic pressure changes under drought stress. The calcium sensor *CBL1* embedded in lipids was activated to close stomata and reduce water loss against ROS accumulation. *COR15* and *COR47* interacted with the lipid membrane to avoid cell dehydration and response to ABA. Activation of the MAPKK pathway resulted in a higher expression of *RD29* to accumulate more wax as a water protective layer. A thickened waxy layer of leaves resulted in reduced stomata-mediated water loss and consequently, *GDSL* elevated drought resistance in plants.

## Conclusions

In this study, a total of 103, 103, 99, 198, 203, 239, 249, and 215 *GDSL* genes were identified in three diploid cotton (*G. herbaceum*, *G. arboretum,* and *G. raimondii*) and five allotetraploid cottons (*G. hirsutum*, *G. barbadense*, *G. tomentosum*, *G. mustelinum,* and *G. darwinii*), respectively. The presence of *cis*-acting elements and up-regulated expression of the *GhirGDSL genes* under drought stress showed that *GDSL* genes have the potential to induce drought stress resistance in cotton seedlings under water deficit conditions. Moreover, over-expressing, the *GhirGDSL26* gene in *A. thaliana* increased germination and survival rates, longer root length, lower ion leakage, and expression of various stress-responsive genes under drought stress. This can explain why plants with overexpressed genes had a higher drought tolerance potential compared with wild plants. Drought resistant potential of virus-induced gene-silenced cotton plants was significantly reduced relative to WT plants with the evidence of more MDA and H_2_O_2_ contents and lowered levels of SOD activity and proline content in the VIGS plants. These data will provide fundamental information and a new candidate gene for improving drought stress tolerance in cotton plants.

## Material and methods

### Identification of *GDSL* genes

The *GDSL* conserved domain PF00657 was used to inquire about proteins in cotton. The *GDSL* protein sequences for the *G. herbaceum* (A_1_), *G. arboretum* (A_2_) and *G. raimondii* (D_5_), *G. hirsutum* (AD_1_), *G. barbadense* (AD_2_), *G. tomentosum* (AD_3_), *G. mustelinum* (AD_4_), and *G. darwinii* (AD_5_) were downloaded from CottonGen (https://www.cottongen.org/) [60]; *Theobroma cacao* and *Oryza sativa* was obtained from phytozome (https://phytozome-next.jgi.doe.gov/pz/portal.html). *Arabidopsis thaliana* was downloaded from TAIR (https://www.arabidopsis.org/) using the Blastp program. The result sequences were further analyzed with Pfam (https://pfam.xfam.org/) [61] and SMART databases (http://smart.embl-heidelberg.de/) to ensure the presence of the *GDSL* domain.

### Multiple sequence alignment and phylogenetic analysis

A multiple sequence alignment of the full-length amino acid sequences of putative GDSL members from different plants was performed by MAFFT version 7 (MAFFT for Windows—a multiple sequence alignment program (cbrc.jp). The phylogenetic tree was constructed using the maximum likelihood method by FastTree.exe (FastTree—Bioinformatics Workbook). iTOL online instrument (iTOL: had.nwk (embl.de) was used to visualize and beautify the phylogenetic tree. Moreover, the isoelectric point and molecular weight of deduced GDSL proteins were predicted online by ExPASy Server (http://www.expasy.org/tools/).

### Chromosomal allocation, gene structure analysis, and motif identification

TBtools was used to examine the chromosomal distribution of *GDSL* genes on cotton chromosomes according to gene position [62]. The gene structure and position of the introns and exons were performed using a gene feature visualization server GSDS 2.0 (http://gsds.gao-lab.org/). The conserved motifs of the *GDSL* genes were identified by the online tool MEME motif suite (https://meme-suite.org/meme/index.html). The conserved domain information of the *GDSL* was determined through the Conserved Domains Database (CDD) (https://www.ncbi.nlm.nih.gov/cdd/).

### *Cis-*element identification and Subcellular localization prediction

The nucleotide sequences 2000 bp upstream of the transcription start site of the *GDSL* genes for *G. hirsutum* were extracted from the Cotton FGD database (https://cottonfgd.net/) and submitted to PlantCARE website (http://bioinformatics.psb.ugent.be/webtools/ plantcare/ html/) to ascertain the associate cis-regulatory elements in the promoter region [63]. The subcellular localization prediction of all GDSL proteins was performed using the online tool WoLF PSORT (https://wolfpsort.hgc.jp/) [64].

### Plant material and treatments

In this study, the seeds of *Arabidopsis thaliana* ecotype Columbia-0 (N22625) were originally purchased from NASC (https://arabidopsis.info/) and preserved in our lab*.* All the cotton germplasm resources used in this research were preserved in the National Wild Cotton Germplasm Resources Nursery (Sanya, China). All plant resources used in this research were identified by Hou yuqing. The methods applied in the identification of plant resources followed the information described below.

*Arabidopsis thaliana* ecotype Columbia (Col-0) was used to generate the transgenic lines from T0 to T3. The sterilized seeds were planted on a 1/2 MS medium in the following conditions (22ºC, 16 h light/8 h dark). Germinated seeds were then transplanted into a nutrient bowl having nutrient soil and vermiculite mixed in a 1:1 ratio. Four-week seedlings of WT and T3 generation plants were exposed to natural drought stress for two weeks, and then the survival rates of plants were counted after rehydration. To test the germination rate of the transgenic lines and wild type under drought, seeds of three overexpressed T3 generation plants and WT were sown in the 1/2 MS medium containing 200 mM mannitol. The germination rate of overexpressed lines and wild type were recorded under simulated drought conditions. The square plastic dish was divided into four equal parts, and 30 seeds of WT and the three transgenic lines were planted in each part. After seven days, root morphological traits were measured. Three biological replicates and three technical replications were carried out to minimize errors.

Marie Galante-85 (*G. hirsutum* race) (Accession number NB190232), a semi-wild accession with a high tolerance to abiotic stresses was used in the virus-induced gene silencing (VIGS) experiments. Healthy seeds were sown in sterile sand and water. Sulfuric acid-delinted cotton seeds were grown in the germinating box with 40% humidity sterile sand and kept in dark at 28 °C for three days. Seedlings were transplanted to a hydroponic container with Hoagland’s nutrient solution under well-controlled conditions (28 °C in the day and 25 °C at night and 16 h/8 h light/dark cycle) until the expansion of the third true leaf. At the three-leaf stage, cotton seedlings were treated with 16% PEG6000 solution to induce drought stress. Leaf samples were collected at 0, 24, 48, and 72 h post-stress exposure. These samples were immediately transferred into liquid nitrogen and stored at -80 °C for further analysis.

### RNA extraction and RT-qPCR analysis

Total RNA from each sample was extracted using an RNA prep Pure Plant (Tiangen, Beijing, China) kit according to protocol. RNA quality and purity were assessed by 1% agarose gels and a Nanodrop 1000 spectrophotometer. Total RNA with a 260/280 ratio between 1.8 and 2.1 was reverse transcribed using an EasyScript First-strand cDNA Synthesis SuperMix kit (Transgene, Beijing, China). Primer Premier 5 software was used to design primers for all genes (Table S4). The cotton *GhActin* gene was used as a reference gene for RT-qPCR analysis. PCR reaction was carried out in a final volume of 20 μl volume using an SYBR Green master mix following the manufacturer’s instructions. The reaction was performed in the ABI7500 thermal cycler (Applied Biosystems).

### Vector construction and plant transformation

A pBI121-*GhirGDSL26* recombinant vector was constructed with gene-specific primers. The forward primer sequence ‘ACGGGGGACTCTAGAGGATCCATGGACGCCCAAAGCTTTCTCTTCTCAGTA’ and reverse primer ‘CGATCGGGGAAATT CGAGCTCTCAGTCAATAAATTGCTTGAGAGTGTTCTG’, and then transferred into the competent cells of *Agrobacterium tumefaciens* GV3101 using the freeze–thaw method. *A. thaliana* (Col-0) plants were transfected by the floral dip method with modifications [65]. Transfected lines were selected after germinating seeds on 1/2 MS medium added 50 mg/mL kanamycin for positive selection. At the second generation (T_2_), the expression of the target gene in each transgenic line was checked. Subsequently, the three transgenic lines with the highest expression of *GhirGDSL26* were selected for developing the stable T_3_ generation. The phenotypic and physiological investigations were performed on T_3_ homozygous generation.

### Virus-induced gene silencing

The *GhirGDSL26* gene (1074 bp) coding DNA sequence was downloaded from CottonGen (https://www.cottongen.org/) [60] to design its specific primers. The gene-specific primer sequences are listed in Table S4. To construct a 35S promoter-driven TRV2: *GhirGDSL26* vector, target fragments were amplified from *G. hirsutum* Mg85 and subsequently introduced into the tobacco rattle virus vector (TRV2) via restriction enzymes *BamHI* and *SacI*. The TRV2-PDS vector was also constructed as a visual marker to monitor the silencing efficiency. The recombinant vector was then transformed into the competent cells of *Agrobacterium tumefaciens* LBA4404 strain using the freeze–thaw method. The *Agrobacterium* solution was injected into two expanded cotyledons of a ten-day-old seedlings of Mari-galante 85. Injected seedlings were placed under dark for 24 h and then transferred to normal growth conditions for two weeks. Silenced seedlings were exposed to a 16% PEG6000 drought stress. Leaf samples were collected before drought treatment and 48 h post-stress treatment to assess physio-biochemical attributes.

### Estimation of Physiological, biochemical parameters and histochemical staining

Variations in aboveground fresh weight (VAFW), Ion leakage (IL), Excised leaf water loss (ELWL), and Relative leaf water content (RLWC) were measured and determined according to the (Cai et al., 2019) method. Malondialdehyde (MDA), proline (PRO), superoxide dismutase (SOD), and hydrogen peroxide (H_2_O_2_) contents were measured using relevant detection kits followed by the manufacturer’s instructions (Solarbio, Beijing, China). The H_2_O_2_ content in Arabidopsis leaves was assessed using DAB chromogenic kit (Nanjing Jiancheng Bioengineering Institute, Nanjing, China). Leaf cell damage was determined by the trypan blue staining method [66].

### Expression profiling of stress-responsive genes.

The expression of six stress-responsive genes *COR15*, *COR47*, *RD29*, *SOS2*, *CBL1,* and *KIN1* in the overexpressed Arabidopsis plants and wild plants was assessed by RT-qPCR. *AtActin2* was used as endogenous control. The specific primers were designed using Primer Premier 5 software (Table S4). RT-qPCR reaction was run in a 20 μl volume using AceQ Universal SYBR Green qPCR Master Mix (Vazyme, Nanjing, China) and then performed on an ABI7500 thermal cycler (Applied Biosystems).

### Statistical analysis

All data were analyzed using SPSS software. Differences among transgenic lines or VIGS-cotton plant and wild type under treatment and CK conditions were tested using Student’s *t*-test at 0.05 probability level. Means with different lowercase letters show a significant difference. Error bars of standard error in each graph represent the dispersion degree of three replicates.

## Supplementary Information

**Additional file 1.**

**Additional file 2.**

**Additional file 3.**

**Additional file 4.**

## Data Availability

The related gene sequence files of all cotton were downloaded from CottonGen (https://www.cottongen.org/). Sequences of cacao and rice were obtained from phytozome (https://phytozome-next.jgi.doe.gov/pz/portal.html). Arabidopsis thaliana was downloaded from TAIR (https://www.arabidopsis.org/). All data generated or analyzed during this study are included in this article and its supplementary information files. The datasets used and analyzed in this study are available from the corresponding author upon reasonable request.
